# Suppression of Low-Frequency Tones in the Organ of Corti Vibrations of the Basal Turn in the Mongolian Gerbil Cochlea

**DOI:** 10.1007/s10162-025-01012-0

**Published:** 2025-11-10

**Authors:** Jonathan H. Siegel, Wenxuan He, Tianying Ren

**Affiliations:** 1 Department of Communication Sciences and Disorders, School of Communication, Northwestern University, 2240 Campus Drive, Evanston, IL 60208-2952 USA; 2 Oregon Hearing Research Center, Department of Otolaryngology, Head and Neck Surgery, Oregon Health & Science University, 3181 SW Sam Jackson Park Road, NRC04, Portland, OR 97239 USA

**Keywords:** Intracochlear vibrations, Reticular lamina region, Basilar membrane, Mongolian gerbil, Otoacoustic emissions, Two-tone suppression

## Abstract

**Purpose:**

The basilar membrane (BM) motion evoked by single or two-tone stimuli shows nonlinearity largely confined to the region of the traveling wave peak(s) with a passive and linear response basal to the peak. For the same stimuli, nonlinear two-tone interactions in the ear canal pressure and cochlear microphonics appear to originate in a region that extends considerably basal to the peak of the BM traveling wave. Recent measurements from the reticular lamina region (RL) of the organ of Corti in the mouse apex exhibit active gain and broadly tuned two-tone suppression of the response to a lower-frequency probe stimulus that is not measured in the BM at the same location. These results suggest that suppressible active gain is evident in the RL region motion in the region basal to the characteristic frequency (CF) place of the probe tone. The purpose of this study is to explore the spatial extent of active gain and nonlinearity of the cochlear partition by measuring two-tone suppression in the RL region and BM responses to a probe two octaves lower in frequency than the CF of the recording location.

**Methods:**

In the current study in the basal turn of the Mongolian gerbil cochlea (15 animals), we used a sensitive custom-built scanning low-coherence heterodyne interferometer to measure two-tone interactions between a suppressor tone near the local CF (~ 20 kHz) and a probe tone two octaves below CF in the vibrations of the RL region and BM.

**Results:**

In sensitive animals, we demonstrate prominent two-tone suppression of the response in the motion of the RL region to a 5 kHz 40 dB SPL probe tone for suppressor tones near the ~ 20 kHz CF at levels as low as 40 dB SPL. Suppression of the probe response became more pronounced as the suppressor level was increased. Responses to the 40 dB SPL probe could not be measured in BM where responses were in the noise. When the probe level was raised to 60 dB SPL, such that the BM response was well above the system noise, we did not measure any change in BM vibrations for suppressor levels up to 70 dB SPL.

**Conclusions:**

Both active gain and nonlinearity in cochlear mechanics originate in the organ of Corti at a location that extends far basal to the place of the probe tone and not in the BM. Together with the previous reports by others, we conclude that the nonlinear acoustic interaction in the ear canal between a probe tone and tones much higher than the probe frequency is caused by the reduction (suppression) of mechanical gain actively generated by outer hair cells (OHC) in the organ of Corti. There was no indication that the suppressor generated a response at the probe frequency that was not already present in the response to the probe tone presented alone. Our results support the hypothesis that stimulus-frequency otoacoustic emissions (SFOAEs) measured in the ear canal sound pressure originate in a large region extending basal to the place of the probe tone. The BM does not appear to be the site of either generation or propagation of SFOAEs.

## Introduction

Vibrations of the basilar membrane (BM) show physiologically vulnerable gain and nonlinearity for frequencies near the characteristic frequency (CF) [1]. These attributes are not evident for frequencies below about 0.6 × CF, for which the response appears linear. The response of the BM to stimulus tones near CF can be suppressed by a second tone near or above CF with frequencies extending to no more than one octave above CF. Corresponding to the linearity of the response to single tones well below CF, the response of the BM to tones lower than ~ 0.6 × CF is not suppressed by a second tone of any frequency for stimulus levels at least as high as 100 dB SPL [2]. Nonlinear two-tone interactions, including suppression and the generation of intermodulation distortion in BM vibrations, appear to require overlap between the peak regions evoked by the stimulus tones (e.g., [3, 4]).

On the other hand, the mechanics of the organ of Corti at comparable frequencies below CF are neither passive nor linear. The development of optical methods that can measure vibrations both in BM and the organ of Corti in living cochleas, including Optical Coherence Tomography (OCT) [5] and heterodyne low-coherence interferometry [6] is a new tool that has greatly enhanced the study of intracochlear mechanics. Studies performed in several labs using these optical techniques have independently demonstrated vulnerable gain and nonlinearity in organ of Corti vibrations below CF that are not seen in the BM, disputing the long-held notion that these mechanical phenomena are only present near CF [7–20] The gain in organ of Corti vibrations below CF in a sensitive animal appears only mildly compressive over more than 40 dB range of stimulus level, saturating at levels above ~80 dB SPL that presumably reflects saturation of the hair cell transducer (e.g., [15, 19, 20]). The near-linear large gain exhibited in the organ of Corti at lower levels represents outer hair cell (OHC)-mediated amplification, as it decreases dramatically postmortem. This vulnerability likely requires that normal ionic and voltage driving forces are necessary to power OHC-mediated gain. Moreover, the spectrum of intermodulation distortion products in the RL is reported to be considerably broader than that measured in the BM of Mongolian gerbils [4]. This is similar to the spectrum measured in the RL vibrations [21].

Two-tone suppression of otoacoustic emissions in the external ear canal has been well documented in the literature. Guinan had discovered that the response to a low-level probe stimulus tone could interact with a second tone with a much higher frequency, likely indicating suppression of a stimulus-frequency otoacoustic emission (SFOAE) evoked by the probe tone [22]. Motivated by this discovery, Siegel and colleagues made extensive measurements in the ear canals of chinchillas that suggested that the region of generation of SFOAEs was large and extended considerably basal to the CF place of the probe tone [23]. Other studies confirmed and extended these findings [24–26]. However, two-tone nonlinear interaction had not been measured in the BM response under conditions similar to those for the ear canal acoustical experiments, and the basis for the nonlinear two-tone interaction in regions basal to the place of the probe tone was questionable. Suppressor tones near the frequency of a low-level probe tone are commonly used to separate the stimulus tone from the SFOAE generated in the cochlea [27–30].

Several cochlear models have predicted nonlinear two-tone interactions on the BM basal to the peak region [31–33]. One of these models proposes that suppressors acting basal to the place of the probe induce a nonlinear response to the probe that is not present when the probe tone is presented alone [31, 34]. Following the logic of these models, if the cochlear region basal to the peak is perfectly linear, then adding a suppressor tone would have no effect on the probe tone in this region. But if this basal region is nonlinear, and the suppressor tone drives this region into its nonlinear range, then the local impedance could change, and reflection of the probe tone might occur from the region where the suppressor tone induces nonlinearity. Assuming that these nonlinear reflections of the probe reach the ear canal, they would appear as a contribution to the emitted pressure at the probe frequency but with a relatively short delay compared with reflections from the peak region [32, 33], but no experimental evidence supports these model predictions. Presumably, if the model predictions were valid, it would be possible to measure in the BM, at some intracochlear location basal to the place of the probe, vibrations driven by the probe that increase in amplitude (or simply change) with the addition of a suppressor tone.

On the other hand, if the suppressor consistently reduces the amplitude of the existing basal response to the probe measured in the BM or organ of Corti, then the two-tone interaction in the ear canal would plausibly result from the reduced intracochlear vibration at the probe frequency resulting from suppression. The problem with both of these proposed explanations for nonlinear interaction between tones widely separated in frequency is the absence of evidence of obvious nonlinearity in BM vibrations far basal to the place of the probe tone. Neither the study by Siegel and colleagues [23] nor the one by Shera and colleagues [34] has been published. A similar, likely related, controversy arose over evidence for an extended spatial origin of distortion product otoacoustic emissions (DPOAEs) that extended unexpectedly far basal to the places of the two stimulus tones [35–37]. As expressed eloquently by Charles Molnar: “Nothing so resembles an artifact as a new phenomenon.” Some critical information needed to resolve the controversy was missing.

Studies with particular relevance to this paper have reported suppression of the amplitude of the RL region response for suppressors acting basal to the place of the peak response to a low level probe when nonlinear interaction is not observed in the BM [10, 13, 19]. Furthermore, Charaziak showed that this basal mechanical suppression corresponds to a change in the acoustic response to the probe in the ear canal [7]. So far, the Charaziak study in the apical turn of the mouse cochlea is unique in demonstrating a direct correspondence between intracochlear suppression of RL region motion and the presence of nonlinear two-tone interaction in the ear canal. Measurements of this kind are needed from different cochlear regions and species to establish whether this finding applies generally. But we now have plausible explanations for the two-tone interactions measured in the ear canal more than 20 years ago and to also evaluate alternate explanations provided by cochlear models.

The goal of this study is to explore the spatial extent of nonlinearity of the cochlear partition by measuring two-tone suppression in the RL region and BM responses to a probe two octaves lower in frequency than the CF of the recording location, using a custom heterodyne low-coherence interferometer[4, 20]. Suppression of the response measured in the motion of the RL region was consistently observed in animals with high sensitivity, but not in the BM for suppressors up to 70 dB SPL. Since this suppression of the response to a low frequency probe tone was measured at a high-frequency location, our results indicate that the SFOAE arises in a large region that extends far basal to the place of a low-level probe, likely through amplification by OHC in the basal region.

## Methods

Fifteen young healthy Mongolian gerbils of both sexes aged 5–9 weeks (40–80 g) were used in this study. The animal use protocol was approved by the Oregon Health & Science University Institutional Animal Care and Use Committee (IP000000932). Data reported in this paper were collected from ten sensitive cochleae. Results from five animals were excluded because of an insensitive cochlea at high frequencies, poor signal-to-noise ratio, and/or incomplete data sets. Cochleas were judged to be sensitive based on the presence of compressive nonlinearity and sharp tuning near CF, distortion product otoacoustic emission (DPOAE) levels with less than 5 dB reduction below the average for other sensitive ears. An additional criterion, the presence of RL region two-tone suppression, was added from the results of this study. The RL region suppression disappeared with even small reductions in sensitivity.

Measurement of the cochlear partition vibrations. Experiments were conducted on a vibration isolation table inside an acoustically attenuated and electrically shielded double-wall booth. Under anesthesia induced by ketamine and xylazine (100 mg per kg and 10 mg per kg intramuscularly), a tracheotomy was performed and natural free breathing was maintained. Body temperature was kept constant at ~ 38 °C using a heating blanket that was feedback-controlled with a rectal temperature probe. The animal’s head was held firmly using a custom-built head holder mounted on a computer-controlled three-dimensional translational stage with rotation capability. A ventrolateral surgical approach was used to expose the left bulla and to transect the external ear canal. An acoustic probe connected to two speakers and a microphone was coupled to the remaining bony ear canal to form a closed sound field. A 5 kHz tone (probe) at either 40 or 60 dB SPL (0 dB SPL = 20 µPa) was presented to the ear canal, and a second tone (suppressor) was presented simultaneously, varying in frequency between 0.5 and 30–35 kHz with a constant level from 40 to 80 dB SPL. The spectrum of the resulting response was displayed on a dynamic signal analyzer (SR785, Stanford Research Systems, Sunnyvale, CA) and recorded through a digital lock-in amplifier (SR830 DSP, Stanford Research Systems, Sunnyvale, CA).

The anterior and lateral bony walls of the bulla were removed using a sharp blade to visualize the cochlea and the round window. About one-third of the round window membrane was removed with a tungsten hook, and the opened area was covered with a glass coverslip. Great care was taken to avoid bleeding by preserving blood vessels on the round-window membrane. When the BM was positioned approximately in the horizontal plane, a white light beam through a single-mode optical fiber was brought close to the lateral bony wall of scala vestibuli and scala media. The position and angle of the optical fiber were adjusted so that landmarks of the cochlear partition were visible through the microscope. Under direct visualization, low-coherence light from the object arm of the interferometer was focused on the center of the outer hair cell region through an infinity-corrected long working distance objective lens (Plan Apo 20 ×, NA 0.42, Mitutoyo, Japan). A custom-built scanning low-coherence heterodyne interferometer was used to measure vibrations inside the cochlear partition. This instrument has unprecedented sensitivity, temporal resolution, and spatial resolution for measuring micromechanical vibrations in living cochleae [6]. The locations of the BM and RL region were indicated by the carrier signal level as a function of the transverse location and confirmed by the magnitude and phase of the cochlear partition vibration [20]. After the BM and RL region locations in the transverse direction were determined, the object light beam of the interferometer was focused on those locations sequentially for vibration measurements.

Two-tone suppression was measured using a fixed 5 kHz probe tone at either 40 or 60 dB SPL and a suppressor tone varied in frequency in steps between 0.5 and 35 kHz at 40, 50, 60, 70, and 80 dB SPL. The unsuppressed response to the probe tone was taken at suppressor frequencies above ~25 kHz, where no suppression was noted for any suppressor level. The data in Figs. 2, 3, 4, and 5 show the probe response in the presence of the suppressor, a direct measure of suppression.

**Fig. 1:**
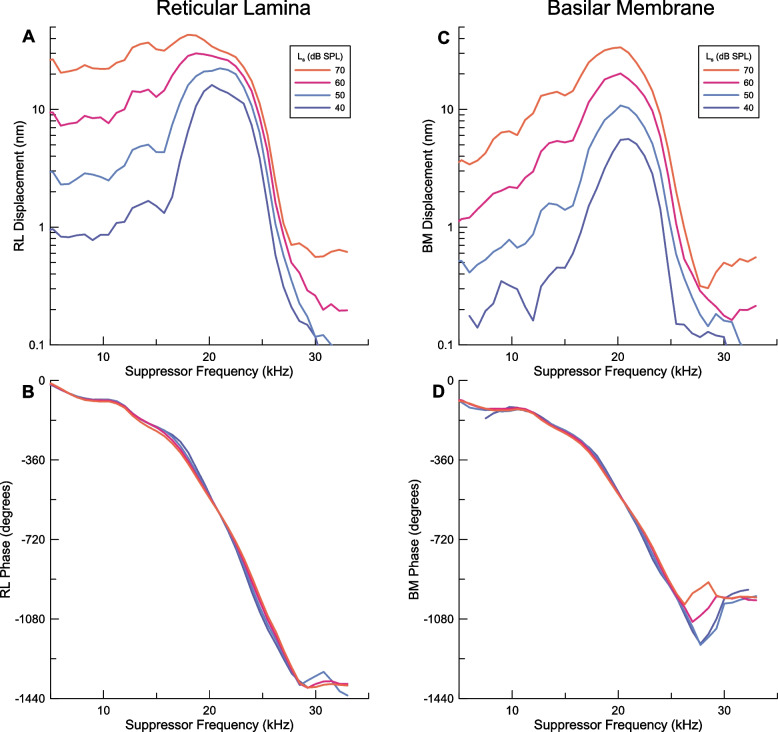
Representative response to single tone stimuli in a sensitive cochlea for stimulus levels of 40 to 70 dB SPL. The CF of the measurement location was ~ 21 kHz. **A** Amplitude and **B** phase of the response of the RL region. This is the most direct measure of the excitation of the outer hair cells that caused suppression. **C** Amplitude and **D** phase of the single tone response of the BM

**Fig. 2:**
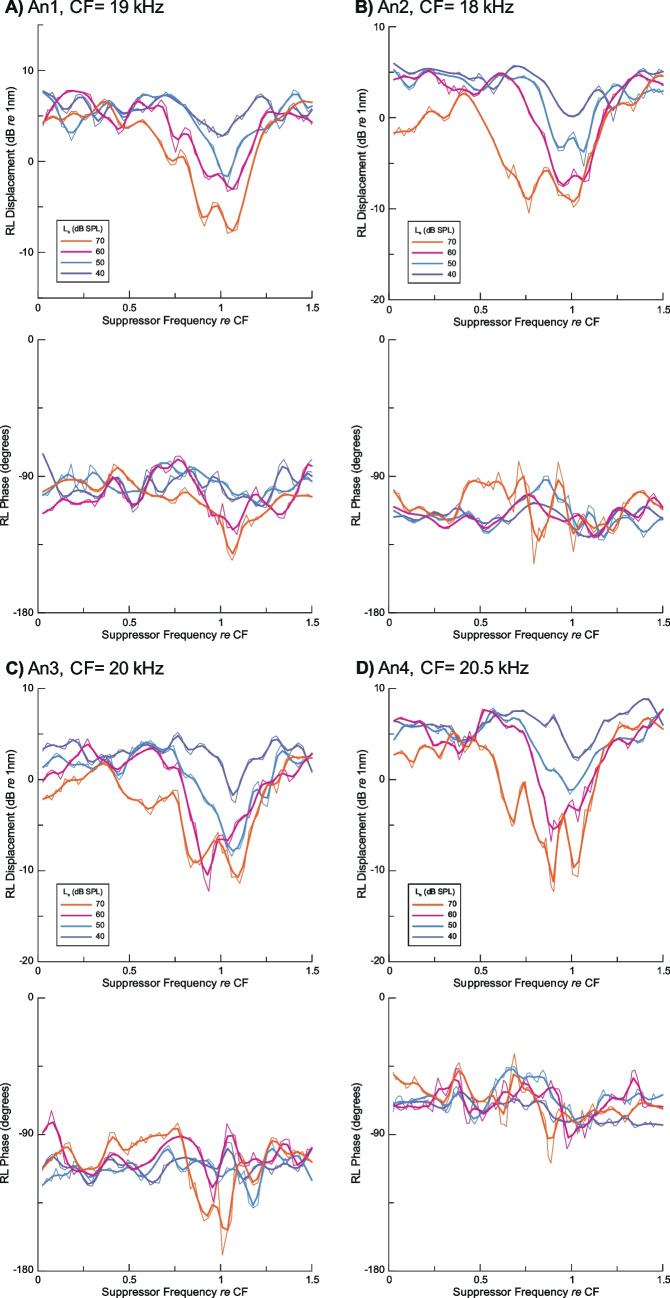
Suppression of RL region active gain by tones with levels (Ls) from 40 – 70 dB SPL for a fixed 5 kHz, 40 dB SPL probe tone measured at a basal location in four sensitive animals with CF noted individually. Suppressor frequencies are normalized to the local CF. Probe response amplitudes (in dB re 1 nm) are smoothed gently using LOESS fits (thicker lines) that also allowed normalized suppressor frequencies to be interpolated to a common frequency range to facilitate averaging in Fig. 3. The unsmoothed data are also shown (thinner lines). For each panel **A**-**D**, the probe displacement amplitude is the upper plot, while its phase is in the lower plot. Suppression is present at all cases even at the lowest suppressor level (40 dB SPL). The phase of the probe tone shows no consistent effect of the suppressor tones

**Fig. 3:**
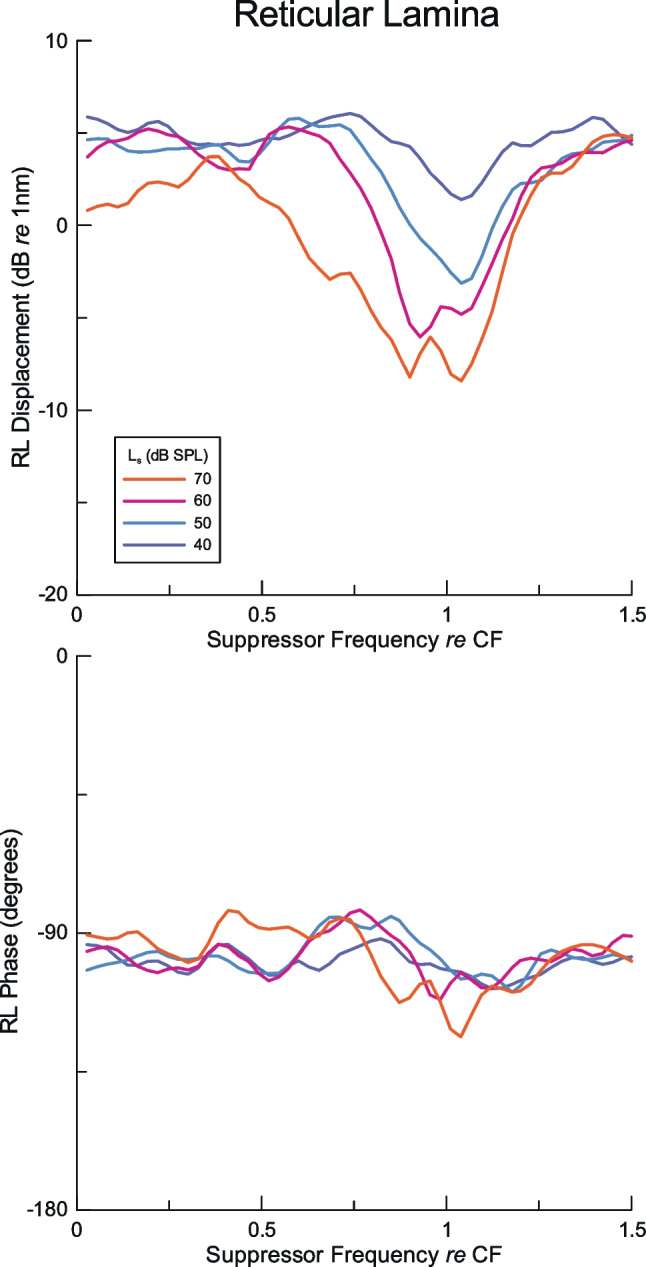
Average of RL region suppression of the fixed probe tone data from Fig. 2. Trends in the averages are evident in the data from individual animals in Fig. 2

**Fig. 4:**
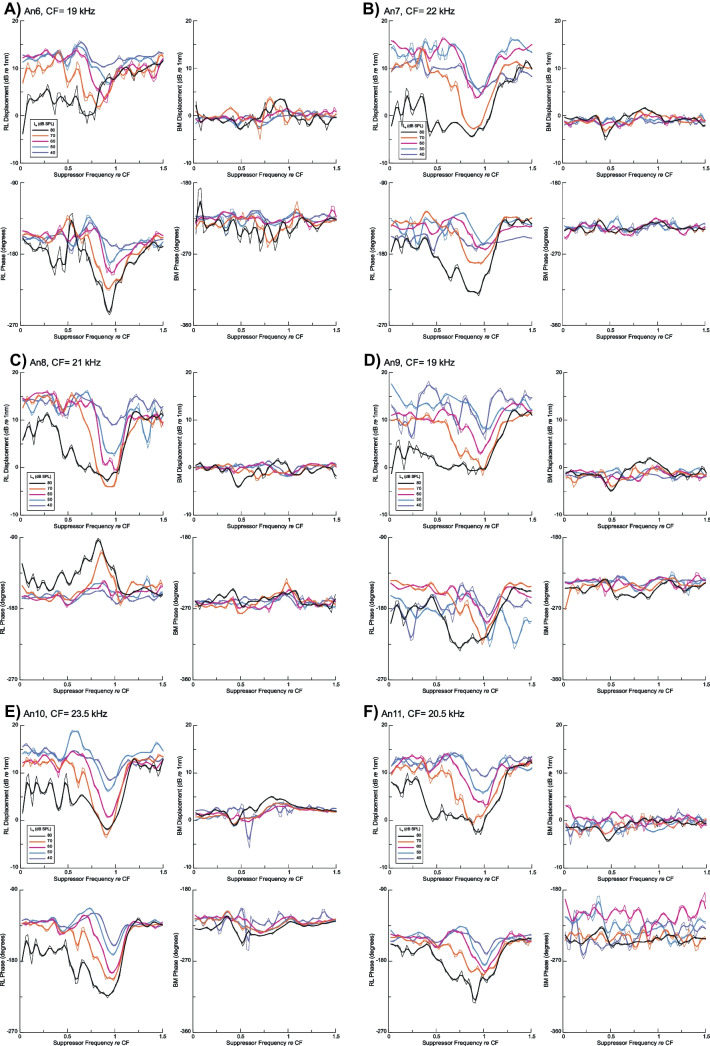
Suppression by tones with levels (*L*_s_) from 40 to 80 dB SPL for a fixed 5 kHz, 60 dB SPL probe tone measured at a basal location in six sensitive animals with CF noted individually. For each **A**–**F**, the RL region probe amplitude (top) and phase (bottom) are on the left while the BM probe response amplitude (top) and bottom (bottom) are on the right. Measurements of BM vibrations at the same location reveal no effect of suppressor tones from 40 to 70 dB SPL, but small changes in the amplitude of the probe response that vary between animals are noted at some frequencies for 80 dB SPL suppressors. In contrast to the data of Fig. 2, the phase of the probe response in the RL region vibrations changed considerably for the 60 dB SPL probe in Fig. 4, but there was no consistent effect of the suppressor on the BM probe phase at the same place

**Fig. 5:**
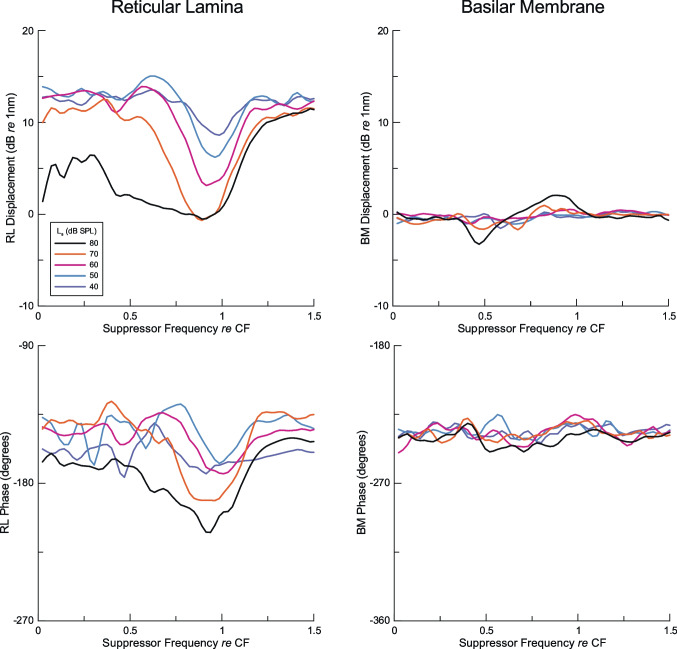
Average of RL region and BM suppression of the fixed probe tone data from Fig. 4. Trends in the averages are evident in the data from individual animals in Fig. 4. Suppression was evident in the RL region probe amplitude response, and phase changes were evident for all suppressor levels (*L*_s_). There was no evidence for amplitude suppression in BM for suppressor levels of 70 dB SPL or lower, but a biphasic change was noted in the BM probe amplitude for the 80 dB SPL suppressor. There was no evidence of a phase change in the BM probe response for any suppressor level

## Results

### Suppression of Responses to Low-Level Probes in the Reticular Lamina Region

A representative example of magnitude and phase responses of the RL region and BM to single tones measured in a sensitive cochlea is shown in Fig. 1. Consistent with previous measurements [17, 20], the displacement amplitude of the RL region vibrations (Fig. 1A) is larger than that of the corresponding BM (Fig. 1C). It shows stronger compression near the CF of the measurement location and progressively greater displacement, relative to BM, as the stimulus frequency is lowered. Correspondingly, the RL region phase (Fig. 1B) shows greater level dependence than BM (Fig. 1D). The RL region response is band-pass at 40 and 50 dB SPL, but trends toward low-pass as the level of the stimulus is raised (Fig. 1A). Although the BM response becomes more broadly tuned at higher stimulus levels, the decline at lower frequencies is apparent. The RL region response is a direct measure of the mechanical displacements that activate the outer hair cells where suppression undoubtedly occurs. It should be noted that, at frequencies near 5 kHz, the increase in the RL region response between 40 and 60 dB SPL was almost exactly linear (10 dB in response amplitude/10 increase in stimulus level within a small fraction of 1 dB). Very slight compression was measured from 60 to 70 dB SPL (~ 9 dB/10 dB), but the reduced slope was not obvious to the eye in Fig. 1A. The RL displacement was ~ 10 nm at 60 dB SPL, so the RL region single tone response in this frequency range at levels below 60 dB SPL can be considered linear.

We applied a probe tone at 5 kHz that was much lower than the ~ 20 kHz local CF, such that its peak response was far apical to the location where measurements were made. It was also important to set the probe level as low as possible while still allowing its response to be measured reliably. The 40 dB SPL chosen probe level produced ~ 2 nm displacement of the RL region near the 20 kHz place, which was about two orders of magnitude above the ~ 0.01–0.02 nm system noise floor [6, 20]. This is also a probe level similar to that commonly used in SFOAE measurements, including those that indicate a contribution to the emission well basal to the CF place of the probe tone [22, 24, 26, 29, 38]. Unfortunately, the BM response amplitude is ~ > 20 dB lower than the RL region response at this location, placing it below the noise floor. A separate set of measurements, described later, used a higher probe level (60 dB SPL; Figs. 4 and 5) where both RL region and BM measurements had good S/N and could be compared directly.

Figure 2 shows results from four sensitive animals that demonstrate that the suppression patterns can be readily observed without averaging across animals. Because the CFs of the four locations differed slightly from 18 to 20.5 kHz, the frequency axis was normalized to the individual CF. This made an additional step necessary to obtain an average. The individual raw data (Fig. 2 thin lines) were smoothed gently using LOESS fits (*α* = 0.082) (thicker lines). The fitting also allowed an interpolation to the same set of scaled frequencies. Suppression of the amplitude of the probe can clearly be seen in all four cases for the lowest suppressor level used (40 dB SPL), indicating that the threshold for suppression was ~ 10 nm (Fig. 1A). Suppression was tuned to the local CF for suppressors at 60 dB SPL and below but was extended to lower frequencies for 70 dB suppressors. This is the only suppressor level that exceeded an RL region displacement of 10 nm for suppressor frequencies below 15 kHz (Fig. 1A). The phase of the probe response does not show consistent effects of the suppressor for the 40 dB SPL probe tone.

The averaged magnitude and phase data based on the individual results in Fig. 2 are presented in Fig. 3, which clearly shows suppressor-induced changes in the amplitude of the RL region probe responses at different sound pressure levels, with the absence of consistent changes in the phase.

### Suppression of Responses to Moderate Level Probes in Reticular Lamina Region and Basilar Membrane

Increasing the level of the 5 kHz probe tone to 60 dB SPL increased the amplitude of response of the BM near the 20 kHz place to ~ 1 nm, an order of magnitude above the system noise floor (Fig. 4). All six animals showed clear suppression of the probe response amplitude (Fig. 4A–F) in the RL region, but not in BM, except at the highest suppressor level (80 dB SPL; Fig. 4A, B, D, only slight suppression in Fig. 4 C, F). These trends are shown even more clearly in the averages calculated to the LOESS fits to the raw data (Fig. 5). As shown in the suppression of the lower-level probe in Figs. 2 and 3, the onset of suppression for the 60 dB SPL probe is ~ 40 dB SPL, corresponding to a suppressor displacement of ~ 10 nm, similar for the 40 dB SPL probe. Suppression remains relatively sharply tuned to the local CF (Fig. 5A) but showed significant broadening to frequencies more than an octave below CF only for 80 dB SPL suppressors. The RL region phase showed suppressor effects for the 60 dB SPL probe tone, in contrast with the inconsistent small phase changes with the 40 dB SPL probe shown in Figs. 2 and 3. On the other hand, no consistent effect of the suppressor was observed in BM responses for suppressors of 70 dB SPL and below, the exception being for the 80 dB SPL suppressor.

## Discussion

The measurements of vibrations of the RL region and BM reported here show clear evidence for two-tone suppression in the RL region but not the BM for suppressor tones near the ~ 20 kHz local CF acting on fixed probe tones at a frequency two octaves below the CF. Only at suppressor levels of 80 dB SPL is there any hint of nonlinear interaction between the suppressor and the probe in the BM, in some animals. In this situation, the motion of the BM to single-tone excitation does not deviate significantly from linear behavior. Our finding of two-tone suppression in the RL region also adds to the evidence from several labs that active gain in the OHC region is significant for probe tones much lower than the local CF and deviates from linear behavior in other ways. Characterizing this phenomenon was a primary goal of this study.

It should be noted that the position within the organ of Corti where active gain and nonlinearity are observed is somewhat variable between studies. Some studies, including this one, report vibrations of the RL or RL region [7, 10, 12, 15–17, 20, 39], while others report that the largest amplified responses are measured in the OHC region [8, 9, 13, 18, 19]. At least part of these differences might be explained by the dramatic changes in the size and submicroscopic structure of hair cells and supporting cells that may constrain motion within the organ of Corti differently, depending on the relative position along the cochlear spiral [40–42].

### Nonlinearity and Active Gain in the Organ of Corti Below CF

Modern optical measurement techniques revealed the presence of vulnerable gain in the OHC region at frequencies where the BM has been characterized as both passive and linear [7, 9–20]. The earliest reports in mice showed level-dependent gain below CF, trending toward linear scaling at the lowest stimulus levels [16, 17]. This trend was also evident in all three turns of the Gerbil cochlea [12]. However, other reports demonstrated that gain below CF scaled almost linearly with stimulus level over a widely varying range of intensity [15, 19, 20, 39]. The gain dropped dramatically postmortem, approaching the gain of the BM in both alive and postmortem states, demonstrating that gain in the OHC region below CF in living animals was active [12, 17, 20]. It was noted later that the gain in the RL region at frequencies well below CF (below 10 kHz) in gerbils was reduced considerably at the highest intensity (80 dB SPL), while linearity was demonstrated in stimulus pressures varying over 5 orders of magnitude, between 20 and 70 dB SPL [15]. While the drop in high-level gain is likely due to saturation of hair cell transduction, it is surprising that the range of stimulus levels where near-linear gain below CF varies so much between experiments, even within the same species.

Level-dependent gain was also reported in so-called hot spots within the organ of Corti [9], but this study used a multitone complex instead of fixed or swept single tones. However, other reports of gain in the OHC region using single-tone stimuli revealed approximately constant gain below CF in the organ of Corti that became more strongly level-dependent during stimulation by a multitone complex [13, 18, 19]. In hindsight, and in view of the results presented here, the multitone stimulus appears to have resulted in mutual suppression in the responses to components of the stimulus, so the single tone results are not equivalent to those obtained with the multitone stimuli and not equivalent to differences in overall stimulus level. These measurements instead provided some of the first evidence of suppression in the motion in the OHC region below CF. More recently, suppression of vibrations below-CF in the OHC region by higher frequency suppressor tones has been demonstrated directly [7, 10]. Additional recent evidence for nonlinearity in the active gain below CF comes from its reduction to near postmortem values in the presence of an intense fixed suppressor tone [19]. The appearance of linear amplification over a range of lower-level stimuli suggests an active quasilinear mechanism at low to moderate stimulus levels. Strimbu and Olson have come to a similar conclusion [19]. However, active gain in the RL region far below CF is reported to saturate and decline toward postmortem passive values for very high stimulus levels in the mouse apex [39]. This shows that the drop in gain to near passive levels does not require either death or irreversible damage to hair cells.

Given that transduction in OHC is demonstratively nonlinear, modeled by Boltzmann functions [43, 44], and that intracellular receptor potentials exhibit harmonic and intermodulation distortion at low to moderate stimulus levels [45], it is puzzling that OHC could produce such near-linear mechanical gain. The situation is reminiscent of operational amplifiers that produce significant gain with distortion reduced through negative feedback. How might such negative feedback happen in the organ of Corti?

The onset of suppression documented in this paper is approximately 40 dB SPL. Likely related is the observation that intermodulation distortion is detected in RL motion for equal level *f*_1_, *f*_2_ stimuli as low as 40 dB SPL in gerbils [4]. Harmonic distortion and a dc component appear at even lower stimulus levels in the RL region, with consistently lower distortion in BM [46]. While the mildly compressive gain in the OHC region below CF in several reports gives the impression of quasilinear growth, this contrasts with the much stronger compression measured near CF. The appearance of harmonic and intermodulation distortion at low levels, along with two-tone suppression, is much more sensitive measures of deviation from linearity than amplitude scaling. The growth of these sensitive measures of nonlinearity is thus consistent with a mildly compressive extended level range of active gain before strong saturation is measured as seen at the local CF in both the OHC region and BM. This observation indicates that both the active gain and various aspects of nonlinearity seen in the OHC region emerge from the physiology of the outer hair cells and the appearance of these phenomena in BM vibrations represents a filtered version of the nonlinear behavior of the hair cells in the organ of Corti.

Active gain at locations far from the resonant CF place is a recent discovery but has a theoretical basis in hair cell models that generate more mechanical energy than needed to counteract damping [47, 48]. This concept is important in recognizing that cochlear amplification does not necessarily depend on an underlying passive mechanical resonance near the CF place. This is clearly the case for sub-CF active gain demonstrated in experiments. The especially rapid recovery of this gain below CF following furosemide administration [49] shows that it requires only functional outer hair cells and a reasonably normal transepithelial driving force, which is the sum of the endocochlear potential and the potassium ion concentration gradient between the endolymph and perilymph.

There is also a theoretical explanation for the difference in two-tone suppression in the RL region vs. the BM we have observed in measurements. A plausible explanation of our experimental findings is provided by a 3D model of the cochlea showing two-tone suppression of a tone much lower in frequency than the local CF by suppressors near CF in the OHC region but with negligible suppression in the BM at the same location [50]. The difference between suppression in RL vs. BM in the model is because the stiffness of the BM is much greater than the RL. The active force generated by outer hair cells in the model is applied to both the RL and BM, but the much lower stiffness of the RL allows suppression of the hair cell forces to be expressed.

### Implications for Otoacoustic Emissions

The evidence of basal suppression reported here is important in its own right as a property of active cochlear mechanics, but it has special relevance to the suppressor method commonly used to separate the SFOAE from the stimulus tone at the same frequency that evokes it [24, 26–29, 38], a variant of measurements of the otoacoustic phenomenon discovered by Kemp [51].

We believe that our findings address the missing information from prior studies of cochlear mechanics before the modern era of optical methods that can measure vibrations of the BM and the organ of Corti in living animals. We have confirmed that the vibrations of the BM far basal to the peak show the absence of active gain and nonlinearity, with only slight deviation from linearity for 80 dB SPL suppressor tones (Figs. 4 and 5), which likely represents passive coupling from the organ of Corti where two-tone suppression was pronounced. More importantly, our measurements of RL region motion show strong evidence of nonlinearity in the form of two-tone suppression. As noted earlier, nonlinearity at basal locations was required by both the hypothesis that part of the SFOAE arises far basal to the place of the probe tone [23, 25, 26] and by the alternative hypothesis that basal contributions of SFOAEs arise through nonlinear reflection [31, 34]. The other crucial finding, supported by other labs, is that the organ of Corti shows active gain even for probe tones far below the local CF. Furthermore, this amplified probe response is consistently suppressed by a second tone near CF at 40 dB SPL or greater, and the degree of suppression increases with suppressor level. In contrast, we found no evidence that the suppressor tone generated a response at the probe frequency that was not already present in the RL region motion in the absence of the suppressor [34].

There are no conditions in which a suppressor caused an enhancement of the response to the probe, except perhaps for the BM response to 80 dB SPL suppressors in some of our data (Figs. 4 and 5). The suppressor-induced reflection hypothesis was proposed in BM models [32–34] but does not appear to have a correlate in the living cochlea for suppressor conditions commonly used to measure SFOAEs [25–29, 38]. Thus, the prediction of these models is not supported by experimental evidence from direct measurements of intracochlear mechanics.

One of the reasons to study SFOAEs is that they represent a measure of otoacoustic emission at a single frequency that can be potentially compared with intracochlear mechanical measures of responses to single tones. The more commonly measured distortion product otoacoustic emissions (DPOAE) suffer from being composed of emissions at intermodulation distortion frequencies, arithmetic combinations of the f_1_ and f_2_ stimulus tones. This also includes harmonics of the stimulus frequencies, as well as SFOAE generated by the stimuli.

Determining the relation between intracochlear measurements of mechanics and OAEs measured in the ear canal is a complex problem, primarily because intracochlear measurements such as those we report are typically made at one or a few locations, while OAEs plausibly originate from a broad region of the cochlea despite existing controversy [30, 34].

Given the cochlear frequency-location map, the frequency relationship between the probe and suppressor, the measurement location, and prior SFOAE suppression data, the present results allow us to draw the following important conclusions. First, the basal suppression of the cochlear partition probe response observed in this experiment supports the previous interpretation that SFOAE originates from a broad region basal to the response peak evoked by the probe stimulus [23, 25, 26] This conclusion assumes that two-tone suppression in the RL region relates directly to the changes measured in the ear canal. Our current results are inconsistent with the popular view that SFOAEs are generated through coherent linear reflection (CRF) from the peak region [30]. It must be emphasized that CRF is based on hypothesized random spatial impedance fluctuations in the BM that have never been demonstrated directly in experiments. This hypothesis diverges significantly from the early thinking of Kemp and colleagues that the sources of SFOAEs are more widely dispersed along the cochlea [29, 38].

The other significant finding of our study related to SFOAEs is the absence of evidence for the participation of the BM in generating SFOAE signals or propagating them to the middle ear and to the ear canal. Even for the 5 kHz probe at 60 dB SPL, the suppressor had no significant effect on the levels of the probe response measured in the BM for suppressors of 70 dB SPL or lower (Figs. 4 and 5). This was the case even for suppressors near the frequency of the 5 kHz probe, where the interaction between the suppressor and probe should have been maximized near the place of the probe. Had the BM been the major route of retrograde propagation of the probe signal toward the middle ear, as hypothesized previously [30], then part of the vibration of the BM measured near the 20 kHz place in our data should have changed during the presentation of the suppressor. The OHC region of the organ of Corti appears very likely to be the source of SFOAEs, as vibrations in this region are readily suppressed by external stimuli. Also, the vibrations of the organ of Corti remain large, even far basal to the place of the probe tone, despite decreasing amplitudes of the BM response to the probe tone, likely due to the much greater stiffness of the BM [50].

To connect our intracochlear measurements unambiguously to SFOAEs would require measurements of SFOAEs and intracochlear vibrations in the same animals, in different regions of the cochlea, and in a variety of species during various manipulations such as the application of furosemide [49] or salicylate [52]. Differences in the vibrations of the organ of Corti reported from different labs, species, and cochlear regions suggest that differences in structure may support differing modes of vibration, only some of which may couple effectively to OAEs measured in the ear canal. The hypothesis that suppression in the OHC region invariably leads to evidence of two-tone suppression in the ear canal must be tested directly. But, despite their limitations, OAEs can be used to make measurements of activity as a function of position within the cochlea by varying the frequency or stimuli. Measurements of OAEs can potentially complement intracochlear measurements typically limited to only a few discrete locations.

### What Our Results Tell Us About SFOAE Estimation Using the Suppression Method

The suppressor method typically uses two separate measurements of the pressure response in the ear canal: the first with the evoking probe tone by itself and next with the probe tone presented simultaneously with a second (suppressor) tone at a higher level and at a different frequency. The change in the probe tone between the two measurements was referred to as the “residual” by Kemp and colleagues [38, 53] (see also [27, 28]). The change in the ear canal pressure only indicates that part of the SFOAE is suppressed but does not indicate the part of the emission that is not suppressed, a fundamental “blind spot” in OAE measurements using suppressors. The estimate of the SFOAE cannot be accurate unless the suppressor completely eliminates the source of the emission at all cochlear locations where it is generated. But this cannot be known from the ear canal measurements alone. Intracochlear measurements of suppression are beginning to reveal how extensive this problem is.

In the case of the data presented here, the active RL region gain near the 20 kHz CF for the 5 kHz, 40 dB SPL probe was not completely suppressed even for 70 dB SPL suppressors, though it did drop by almost 15 dB for suppressors near the local CF (Fig. 3). But because the RL region gain for the lower-frequency probe is broadly tuned spatially, a 70 dB SPL suppressor would only produce a reduction in the active gain in the RL region for the probe over part of the basal region that generates it. To suppress an area larger than the spatial extent equivalent to approximately one octave required a suppressor level of at least 80 dB SPL (Figs. 4 and 5). At this level, small effects of the suppressor were detected in the BM in some animals, but suppression was consistently much larger in the RL region, showing that suppression arises overwhelmingly within the OHC region of the organ of Corti and not in the BM.

### A Mechanical Residual Calculated from RL Region Suppression Data

The controversy over whether part of the SFOAE originates far basal to the place of the probe is because, until recently, there was no plausible mechanism to explain acoustic two-tone suppression studies that support this interpretation. The amplitude of the acoustic residual measured in the ear canal quantifies the magnitude of the change and is, by definition, a positive number. How to interpret the phase of the SFOAE residual is another matter, because neither the mechanism nor the location of the nonlinear interaction between the probe and suppressor was known precisely from the perspective of ear canal acoustics. It is reasonable to assume that the location of the greatest change is the peak of the suppressor’s excitation pattern, a view confirmed in our data, but suppression can be assumed to occur over some finite distance. The greater problem is that the acoustic residual could, in principle, represent either a decrease in an unsuppressed response to the probe tone, as we observe directly in this paper, or an increase, depending on whether the extended region of SFOAE generation or the suppressor-induced probe response hypothesis was correct. So what would our suppression measurements in the RL region, where both the mechanism and place are known, look like when recast as a mechanical residual?

**Fig. 6:**
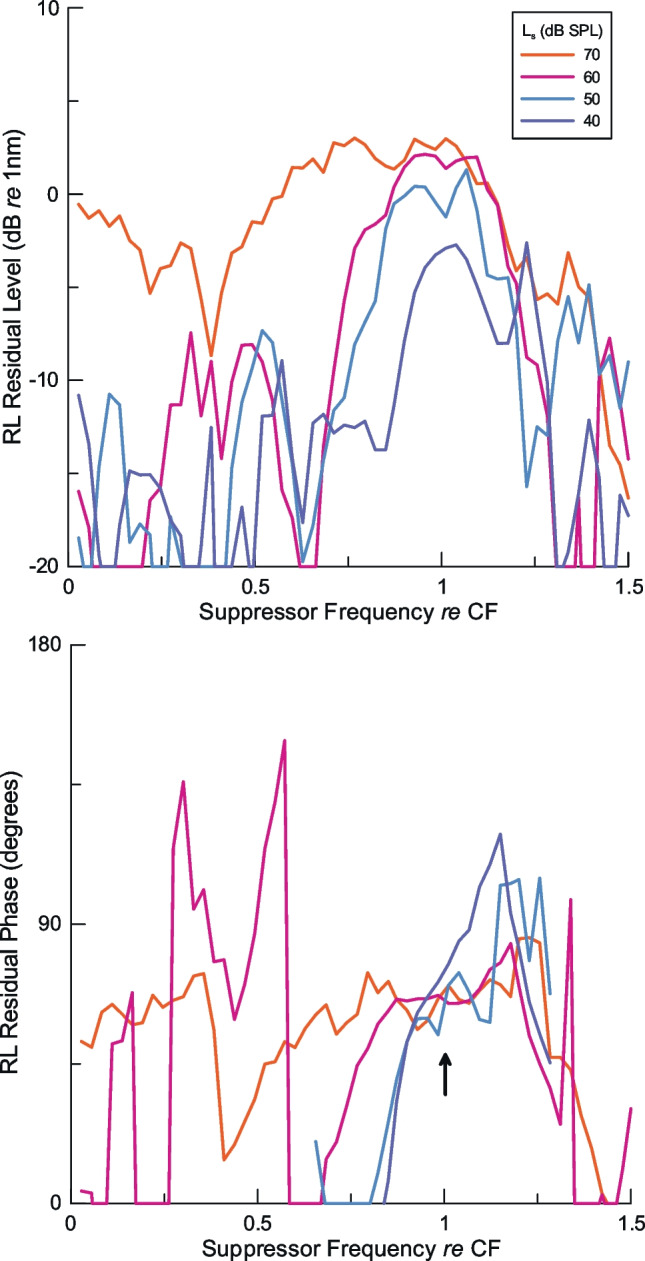
Suppression data from Fig. 2B recast as a residual representing the change in the RL region response to the probe tone from its unsuppressed value. This approximates the situation in the ear canal pressure where the intracochlear source of an SFOAE is not known, only the change observed when the suppressor is presented. In this case, the unsuppressed probe response is measured directly in the RL region vibrations, and the changes are readily appreciated. The phase of the residual converges toward a single value as the level of the suppressor is raised (arrow). Phase data were omitted from responses with the 40 and 50 dB SPL suppressors because large variability made it difficult to see the trends closer to CF

An example of a mechanical residual is derived from the suppression data in Fig. 2B and shown as an equivalent residual in Fig. 6. In this case, the amplitude and phase of the unsuppressed response to the probe tone were not measured, so they were approximated by averaging the probe response with suppressor tones at 40 dB SPL above 1.14 × CF, where there was no clear suppression for any suppressor level. The residual was calculated from the vector difference between the estimate of the probe alone condition and the responses to the other stimuli where the probe and suppressor were presented together. As the level of the suppressor tone increases, the reduction of the amplitude of RL region vibration (~ 1.8 nm unsuppressed) becomes an increase in the amplitude of the mechanical residual (Fig. 6A), while the phase of the unsuppressed RL region response at CF (Fig. 2B), averaged over all four suppressor levels (70.5°), becomes −121.8° in the residual (Fig. 6B, arrow), a change of 192.3°, close to the 180° expected for the suppressed amplitude. Even for suppressor frequencies near the local CF, the amplitude of the residual measured with a 70 dB SPL suppressor is about 1.3 nm, smaller than the unsuppressed response to the probe. Suppressor tones that deviate from the local CF show a diminished residual, demonstrating the restricted range of effective suppression of even a fairly high-level suppressor. For lower-level suppressors, our measurements demonstrate that the mechanical residual represents an even greater underestimate of the unsuppressed probe response.

### Direct Tests of the Existence of a Large Basal Region of SFOAE Generation

The suppression study of Dewey et al. [10] was pivotal in demonstrating in the mouse apex suppression of the response to a probe tone more than an octave below the local CF. Their findings have been confirmed with the corresponding presence of an acoustic SFOAE measured in the ear measured by Charaziak [7]. At present, this is the only example of a direct link between suppression of active gain due to OHC action and a component of SFOAEs in any species or location within the cochlea. The active gain in the organ of Corti measured basal to the location of the CF of the probe tone is uniformly reduced in amplitude postmortem and by suppressor tones. We have provided additional confirmation of this finding in the present paper.

A recent paper claims to have shown strong evidence against basal SFOAE generation [54]. Assuming the validity of the original coherent linear reflection model, the experiment was designed to test the hypothesis that most of the SFOAE originates in the region of the peak evoked by the probe. Perfusing the cochlea slowly from apex to base with solutions of either 150 mM KCl or 20 mM salicylate, it was envisioned that the SFOAE would disappear as the solution front passed the CF place of the probe tone. It was expected that the basal extent of the change in SFOAE would be limited to near and slightly basal to the place of the probe. The main limitation of this experiment is that the SFOAE was separated from the stimulus pressure using a suppressor tone near the frequency of the probe. This choice assumes that the emission arises primarily near and/or slightly basal to the peak of the probe response near its CF place. But the suppressor only removes the part of the emission in the region where it saturates hair cell transducer currents, i.e., in the general region of the place of the suppressor tone. As demonstrated directly in this paper, a moderate-level (i.e., 60 dB SPL) suppressor near the place of the probe tone (i.e., 5 kHz) cannot be assumed to suppress the active gain in the RL region or BM, and thus emission generation, far basal to its own peak (i.e., the 20 kHz place), and so would not readily reveal the presence of basal SFOAE components if they exist. The suppression data in our paper provide a direct estimate of the spatial extent of effective suppression by tones of various intensities. In other words, by using a near-probe suppressor to measure the SFOAE, the experiment was highly biased to reveal emission components located near the place of the probe. The logic of the experimental design of Goodman et al. itself is thus inherently circular and did not critically test the hypothesis.

### Evolving Cochlear Models

The recently developed optical measures of intracochlear mechanics are producing advances in their theoretical implications. A model of the cochlea by Moleti and Sisto that was the first to incorporate RL motion [55] has been successful in reproducing broad band intermodulation distortion similar to the DPOAE interference tone data measured by Martin et al. [35–37]. The Martin group found that the levels and phases of intermodulation distortion products 2*f*_1_-*f*_2_ and 2*f*_2_-*f*_1_ were altered by adding a third tone (referred to as an interference tone or IT), providing evidence for an extended region of DPOAE generation extending far basal to the *f*_2_ place. But the Moleti and Sisto model only found evidence for the IT data in the model’s RL and not in the BM [55]. Like the living cochlea, the model demonstrates nonlinear interactions in the RL over a much broader frequency range than in BM mechanics. The nonlinear interactions in the DPOAE IT studies in ear canal acoustics thus most probably originate inside the organ of Corti. Importantly, this model was not successful in accounting for the IT data of Martin and colleagues in the ear canal, due to relatively weak coupling between the organ of Corti and BM. Such weak coupling appears to be necessary in the real cochlea if the vibrations of the RL and BM show large differences in gain and nonlinearity as shown in recent measurements. The failure of this one-dimensional model in replicating the IT data in the ear canal appears to be that coupling to the middle ear in the model is entirely via retrograde transmission along the BM. So a second propagation mechanism through the cochlear fluids appears to be necessary to account for the IT data of Martin and colleagues.

As mentioned previously, the 3D cochlear model of Samaras and colleagues [50] explicitly accounts for why nonlinearities seen in the organ of Corti are not readily detected in BM at the same location. Like the Moleti and Sisto model, it does not couple organ of Corti vibrations to the cochlear fluids, which may be necessary for the model to produce OAEs in the ear canal. Also, the broad spectrum of intermodulation distortion products measured in the gerbil RL region vibrations reported by Ren and He [4] is much more similar to that measured in OAEs measured in the same species [21] than to the spectrum measured in BM. This may plausibly be related to fluid coupling between the organ of Corti and the middle ear. A similar statement can be made regarding Charaziak’s demonstration between two-tone suppression by tones more than an octave above CF in the RL and suppression seen in the ear canal SFOAE [7]. A recently proposed explanation of the mechanism of the cochlear amplifier is that the pressure directly above the organ of Corti makes a critical contribution to the amplified pressure gradient across the cochlear partition that drives the active gain measured in the BM [56]. The activity of OHCs in the organ of Corti is the likely source of the pressure above the organ of Corti, and this is likely related to the generation of OAE signals.

## Summary and Conclusions

In summary, we have described the suppression of the response to low-level probe tones, most prominently when the suppressor is near the CF of the recording location and around two octaves higher than the probe frequency. Suppression was pronounced in the RL region, but not in BM at the same basal location. While the sensitivity of the RL region to the probe tone was large and was well above the system noise, the response to the probe in BM was much smaller. Even when the probe level was raised such that the BM response was well above the noise, there was no effect of the suppressor on the BM response for suppressor levels of 70 dB SPL or lower. The suppression clearly originated from the organ of Corti. This result establishes the plausibility that two-tone suppression measured in the ear canal for suppressor tones above that of the probe frequency corresponds directly to suppression within the organ of Corti.

The evidence presented here establishes the plausibility that contributions to SFOAEs arise from a broad region of the cochlea and are not confined to the peak generated by the pure tone probe stimulus. We were unable to find any evidence that the BM is involved in either generating SFOAEs or that it is the primary route of retrograde propagation to the middle ear. In particular, we found no evidence for the suppressor-induced contributions to the probe response that were not present in the absence of the suppressor. Instead, the suppressor consistently reduced the amplitude of the RL region probe response at frequencies below the local CF. The optical methods that are revealing essential aspects of cochlear amplification also demand a reevaluation of old models developed before the current era of the study of cochlear mechanics. Previous measurements of OAEs suggesting widespread contributions from basal locations [22, 26] were not compelling in the absence of recent intracochlear measurements and were discounted based on old cochlear models created before the modern era of optical measurements.

Finally, new cochlear models with a primary focus on the mechanisms of the cochlear amplifier are providing critical insights into OAE phenomena. Because OAEs are the product of cochlear mechanics of the living cochlea, a better understanding of OAEs requires a better understanding of intracochlear mechanics and the amplification and nonlinearity resulting from OHC activity.

## Data Availability

Data will be made available on reasonable request.
